# Therapeutic effects of acupuncture plus fire needle versus acupuncture alone in lateral epicondylitis: A randomized case–control pilot study

**DOI:** 10.1097/MD.0000000000015937

**Published:** 2019-05-31

**Authors:** Szu-Ying Wu, Cheng-Nan Lu, Chia-Jung Chung, Chun-En Kuo, Jer-Ming Sheen, Tun-Pin Hsueh, Ching-Chih Chen, Kuo-Wei Bi

**Affiliations:** a Department of Chinese Medicine, Kaohsiung Chang Gung Memorial Hospital and Chang Gung University College of Medicine; b Department of Sports Medicine, Kaohsiung Medical University, Kaohsiung; c Department of Nursing, Meiho University, Pintung; d Institute of Traditional Medicine, School of Medicine, National Yang-Ming University, Taipei, Taiwan.

**Keywords:** acupuncture, fire needle, lateral epicondylitis, tennis elbow

## Abstract

**Background:**

The aim of this study was to compare the short-term and intermediate-term efficacy of acupuncture plus fire needle therapy with that of acupuncture alone in the treatment of lateral epicondylitis (LE).

**Methods:**

Thirty-eight patients with LE who had persisted for at least 2 months were enrolled in this prospective, assessor-blinded, randomized controlled pilot trial. Twenty-one patients were randomized to the acupuncture plus fire needle group and 17 to the acupuncture-only group. The primary outcome was the visual analog scale pain score for the previous 24 hours and the secondary outcomes were the maximum grip strength, Patient-rated Forearm Evaluation Questionnaire score, and Medical Outcomes Study 36-Item Short-form Health Survey score. The values at baseline (pretreatment), at the end of treatment, and at 3 months after treatment were used to assess the short-term and intermediate-term effects of treatment. The data were analyzed using the Chi-square test and *t* test.

**Results:**

Within-group analyses showed better results for acupuncture plus fire needle therapy in the short term and intermediate term. Differences in the severity of pain and secondary outcomes were significant in the intermediate term in the acupuncture group. At the end of treatment, none of the differences in outcome scores were significant, except for maximum grip strength in the affected hand in the acupuncture group. No significant between-group differences in short-term or intermediate-term outcomes were observed.

**Conclusion:**

Acupuncture plus fire needle therapy was effective in the short term in patients seeking improvement of LE. Twelve treatments were effective for relieving pain and improving disability in the intermediate term in patients with chronic LE in both study groups. The findings of the pilot study confirm the feasibility of proceeding to a larger randomized controlled study of the longer-term effects of acupuncture plus fire needle therapy in patients with LE.

## 1 Introduction

Lateral epicondylitis (LE), also known as “tennis elbow,” is the most common diagnosis in patients with elbow pain.^[1]^ The incidence of LE is estimated to be 0.4% to 0.7% per year in general practice and 1% to 3% per year in the general adult population.^[2–4]^ LE is caused by repeated overuse of the wrist extensor muscles, leading to tendinosis, particularly in extensor carpi radialis brevis.^[1]^ Although LE is classically regarded as an inflammatory process, the pathology suggests angiofibroblastic hyperplasia; therefore, many authors consider LE to be a degenerative process in the tendons rather than an inflammatory one.^[5]^ LE can be diagnosed by tests that increase pain, including palpation over the facet of the lateral epicondyle, resisted wrist extension, resisted middle finger extension, and passive wrist flexion.^[5]^ Although many management strategies have been used in patients with LE, including pain-relieving drugs, corticosteroid injections, physiotherapy, elbow supports, shock-wave therapy, platelet-rich plasma injections, and surgery, the optimal treatment is still unclear.^[6,7]^ When conservative treatments are unsuccessful or not accessible for cost reasons, an increasing number of patients consider complementary medicine, particularly acupuncture.^[8,9]^ In a systematic review by Trinh et al,^[10]^ acupuncture was found to relieve the pain of LE effectively, but its long-term efficacy could not be established. Another technique commonly used to treat chronic pain in patients with LE is fire needle therapy, which combines acupuncture and moxibustion. A case report demonstrated a significant short-term effect of fire needle therapy in a patient with LE.^[11]^ Moreover, it has been shown that a combination of rehabilitation and fire needle therapy is effective for at least 4 weeks in patients with LE.^[12,13]^ However, although the early case report demonstrated effects lasting for 4 months, there has been insufficient evidence for the intermediate-term or long-term therapeutic effects of acupuncture or fire needle therapy in terms of reducing the pain associated with LE.^[1,11]^

We hypothesized that the therapeutic effect of acupuncture plus fire needle therapy would be better than that of acupuncture alone. The aims of this pilot study were to investigate whether or not the short-term and intermediate-term outcomes of acupuncture used in combination with fire needle treatment are better than those of acupuncture alone in patients with LE and to determine the feasibility of proceeding to a larger-scale randomized controlled trial investigating the longer-term outcomes of this combination treatment in LE. In this study, we focused not only on the short-term ability of these treatments to improve symptoms but also on the subjective and objective outcomes in the intermediate term.

## 2 Methods

### 2.1 Ethics approval

The study was approved by the human ethics committee at our institution (Chang Gung Medical Foundation Institutional Review Board approval number 98–3818A3). The protocol was registered with ClinicalTrials.gov (Identifier: NCT03820856). Patient confidentiality was ensured before, during, and after the trial.

### 2.2 Study design

This prospective, assessor-blinded, randomized pilot trial was designed to compare the effects of acupuncture plus fire needle therapy with those of acupuncture alone in patients with LE. The patients were recruited from the outpatient acupuncture clinic at Chang Gung Memorial Hospital between January 2010 and January 2011.

### 2.3 Eligibility criteria

All patients identified to have LE during the study period underwent a physical examination to determine their eligibility for enrolment in the study. Patients were selected for inclusion if 2 or more of the following were noted on the physical examination form: pain over the lateral aspect of the elbow; pain on palpation over the lateral epicondyle or the associated myotendinous junction of the common extensor tendon; pain on hand gripping; and pain with either resisted static contraction or stretching of the wrist extensor muscles.^[14]^ Symptoms were required to have persisted for at least 2 months and be unilateral.

The following exclusion criteria were applied: signs and symptoms suggesting a cause other than overuse (e.g., cervical radiculopathy); osteoarthritis of the elbow joints; pathologic, neurologic, and/or vascular findings in the arms; arthritis (local/generalized polyarthritis); radiohumeral bursitis; ligamentous sprain; bilateral tennis elbow; painful shoulder; surgery or dislocation of the elbow; coagulopathy; pregnancy; infection; and malignancy. Patients who had been treated with other therapies or drugs for LE in the 2 weeks before the start of the trial, those who had received corticosteroid injections in the previous 6 months, and those who had already received acupuncture and fire needle therapy for LE were also excluded.

### 2.4 Entry into the study

All patients provided written informed consent before inclusion in the study. The informed consent form was approved by the Chang Gung Memorial Hospital Institutional Review Board for Biomedical Research (IRB#98–3818A3). The patients were informed about the possible adverse effects of acupuncture and fire needle therapy, including bleeding, hematoma, mild pain during needling, local muscle pain, nerve irritation or injury, burning of the skin at the wound site, skin infection, and temporary weakness in the involved muscles. Patients were also informed of rare but serious adverse effects, such as fainting, dizziness, and unstable blood pressure.^[15]^

### 2.5 Randomization

Assignment was based on a predetermined randomization scheme (using a random number table) in a 1:1 allocation ratio. All participants were randomized by use of sealed, opaque, sequentially numbered envelopes.

### 2.6 Study blinding

Blinding of the acupuncturists and patients was not possible because of the nature of the intervention. However, the outcome assessor was blinded to study group allocation (acupuncture or acupuncture plus fire needle). The statistical analyses were performed by a researcher who was blinded to group allocation.

### 2.7 Treatment protocol

All of the acupuncture therapists in the study had at least 3 years of experience, were trained and board-certified in Taiwan, and understood the treatment protocol. The participants were randomly assigned to either the acupuncture group or the acupuncture plus fire needle group and then started their allocated treatment.

### 2.8 Acupuncture group

**Figure 1:**
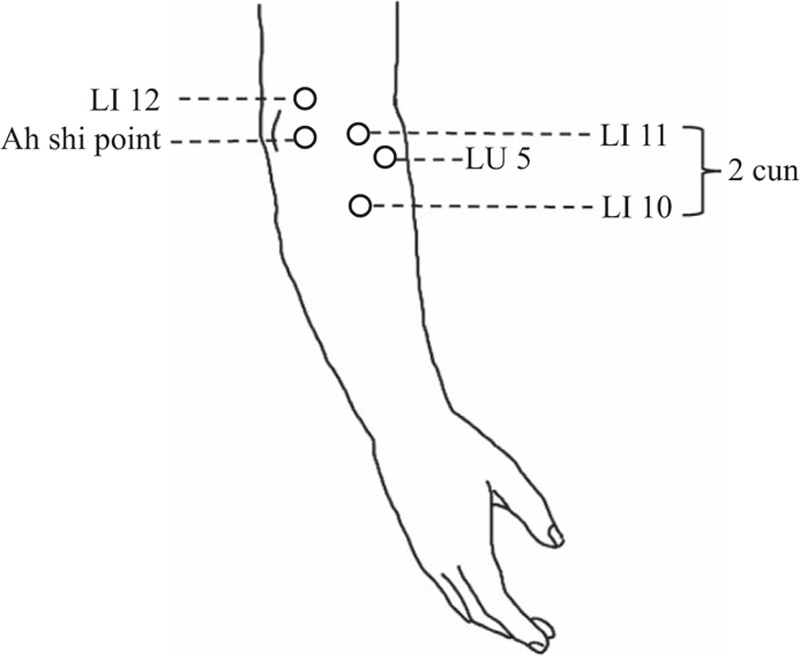
Selection of acupoints.

We selected acupoints that have often been recommended for the treatment of epicondylitis.^[16]^ Triple needling was performed at the Ah shi point, that is, the 1 to 2-cm area encompassing the most painful spot on the humeral epicondyle. We also manipulated acupuncture needles in the following areas: LI10 (Shousanli) and LI11 (Quchi) over the origin of the lateral extensor muscle group of the forearm, with LI10 being 2 cun (a Chinese unit of measurement; 1 cun equals the width of the thumb at the interphalangeal joint) below LI11; LI12 (Zhouliao) comprising an area of 1 cun superolateral to LI11; and LU5 (Chize) in the cubital region (see Fig. 1). Single-use, disposable, sterile, 40-mm, 30-gauge Yu Kuang acupuncture needles (Taipei, Taiwan) were manually manipulated until the patient reported feeling dull pressure and warmth around the needle (De Qi). The LI10 and LI11 acupoints were manipulated using the reducing needling technique, and the other acupoints were manipulated using the reinforcing-reducing maneuver. The needles were left in situ for 15 minutes after manipulation. A triple needling technique was used, whereby 1 needle is inserted deep into the center of the affected area and the other 2 needles are inserted either bilaterally or above and below the first needle. Acupuncture was administered twice weekly for 6 weeks.

### 2.9 Acupuncture plus fire needle group

The protocol used in this group was the same as that used in the acupuncture-alone group except that fire needle therapy was added. The treatments were administered twice weekly for 6 weeks. Fire needle therapy was performed as follows: an acupuncture needle made of tungsten was heated until red-hot over a lighter and then inserted into the 2 Ah shi points (the tender points on the common extensor tendon, specifically extensor carpi radialis). The red-hot needle was inserted rapidly into the Ah shi point (approximately 5–8 fen deep, 1 cun = 10 fen) for approximately half a second and then removed. Pressure was then applied to the needle hole using a sterilized dry cotton ball.

### 2.10 Outcome Measures

Before randomization, a research assistant recorded the baseline demographic data, including sex and age, as well as duration of symptoms. The primary outcome was the visual analog scale (VAS) score for subjective severity of pain within the previous 24 hours. The secondary outcomes were maximum grip strength (MGS), the Patient-rated Forearm Evaluation Questionnaire (PRFEQ) score, and the Medical Outcomes Study 36-Item Short-form Health Survey (SF-36) score. All of the outcomes were evaluated at baseline (pre-treatment), at the end of treatment, and 3 months after treatment to test the short-term and intermediate-term effects of therapy, respectively.

### 2.11 Primary outcome: severity of elbow pain

The severity of the patient's elbow pain was evaluated using a 10-point VAS (0, no pain; 10, most severe pain imaginable) at rest, during motion, and on exertion. Elbow pain lasting more than 24 hours was also evaluated using the VAS.^[17]^

### 2.12 Secondary outcome: functional impairment

MGS was measured using a Jamar Hydraulic Hand Dynamometer (PC5030J1; Preston Corporation, Jackson, MI). The validity of this instrument in diagnosing and evaluating the response to treatment had been established in patients with LE.^[18]^ The patient is required to stand with his or her arm alongside the body with the forearm pronated and the elbow extended while holding the dynamometer. The patient is then instructed to squeeze once with maximum pressure, and the measurement is recorded. The MGS is measured 3 times with a 30-second rest interval between each measurement. The average value is used to maximize reproducibility.^[19]^

The PRFEQ was developed by Overend et al^[20]^ and appears to be useful for assessment of the outcome of treatment for LE. The PRFEQ is divided into 2 sections, that is, pain and function at the elbow. The first section contains 5 items and the second contains 10 items. Each item evaluates the average pain or function of the affected elbow during the previous week. The score ranges from 0 (no pain or difficulty in performing a task) to 10 (the worst pain imaginable or inability to perform a task) for each item. The total score represents the combined score for all questions. The score ranges from 0 to 50 for the pain section and from 0 to 100 for the function section; the value for the function section is then divided by 2 so that 50 is the maximum score. In this study, the Hong Kong Chinese version of the PRFEQ^[21]^ was used to measure functional impairment.

Quality of life was assessed using the SF-36, which was self-administered.^[22]^ The SF-36 measures 8 health dimensions that are divided into 2 broad categories. The first category is a physical component score (PCS) that includes physical function, role limitations related to physical problems, bodily pain, and general health perception. The second category is a mental component score (MCS) that includes vitality, social functioning, role limitations because of emotional problems, and mental health. The raw scores are linearly transformed into a scale of 0 to 100, with higher transformed scores indicating better health. In this study, the Taiwanese version of the SF-36^[23]^ was used to measure general health status.

### 2.13 Statistical analysis

The Chi-square test was used to compare the categorical data, that is, patient sex and elbow affected, between the groups. The independent-samples *t* test was used to compare patient age, duration of symptoms, VAS pain score, the MGS score for the affected hand, and PRFEQ and SF-36 scores between the groups at baseline. To assess the treatment effect, the within-group differences in outcomes were analyzed using the paired-samples *t* test and the between-group differences in scores were analyzed using the independent-samples *t* test. All statistical analyses were performed using SPSS for Windows (version 17.0; IBM Corp., Armonk, NY). A *P* value < .05 was considered statistically significant.

## 3 Results

**Figure 2:**
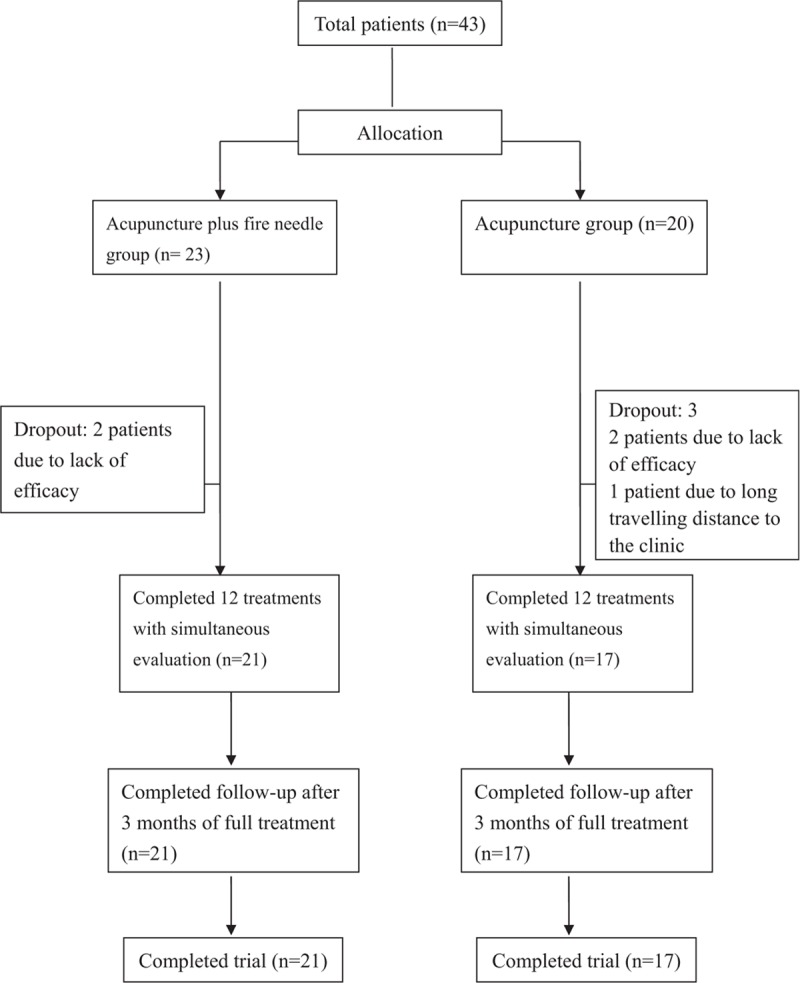
Study flowchart.

Forty-three patients [16 men, 27 women; mean age 48 ± 6.35 (range, 24–58) years] were enrolled in the study (Fig. 2). The mean duration of symptoms was 4.78 (range, 2–12) months. Thirty-three of the 43 patients were right-hand dominant and 10 were left-hand dominant; the dominant hand was affected in 31 patients. The 43 patients were randomly assigned to the acupuncture group (n = 20) or the acupuncture plus fire needle group (n = 23). Two patients in each study group stopped treatment because of lack of efficacy and 1 patient in the acupuncture group stopped treatment because of the long travelling distance to the clinic. These 5 patients did not have post-treatment or follow-up data available so were not included in the analysis. There were no reports of serious adverse effects, such as fainting, dizziness, or unstable blood pressure.

Seventeen of the 38 patients who completed the study were in the acupuncture group and 21 were in the acupuncture plus fire needle group. The demographic and clinical characteristics of the 2 groups are summarized in Table 1. There was no significant between-group difference in patient sex or mean age, duration of symptoms, hand affected, or VAS, PRFEQ, SF-36, or MGS score in the affected hand before treatment.

**Table 1:**
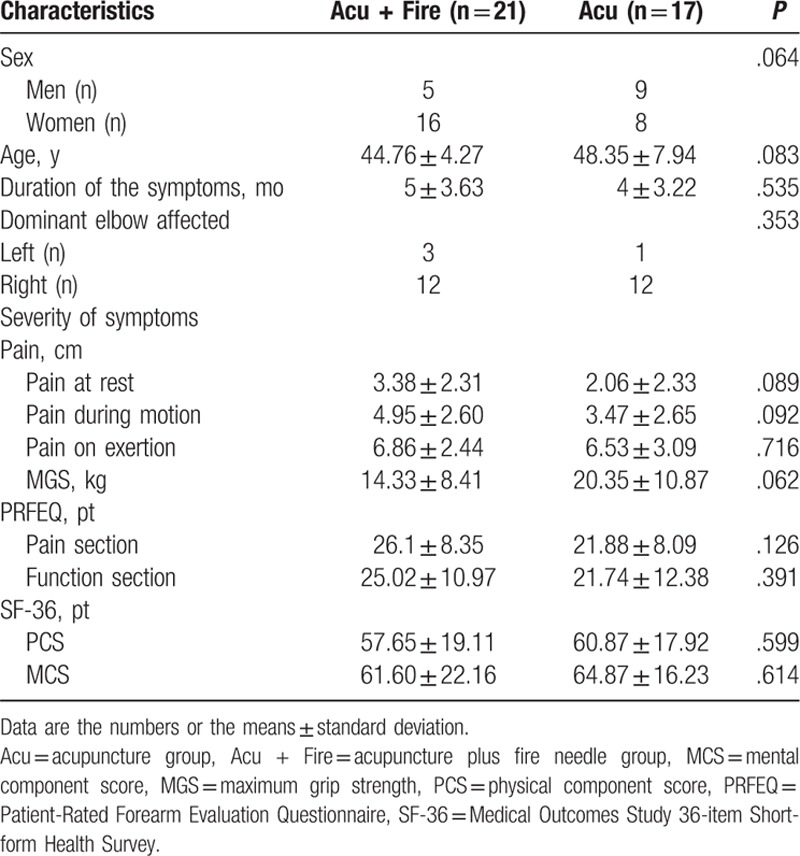
Baseline characteristics of acupuncture plus fire needle group and acupuncture group.

At the end of treatment with acupuncture plus fire needle therapy, there were significant improvements in the VAS scores for elbow pain at rest (*P* < .01) and during motion (*P* < .01) and exertion (*P* < .01). The VAS scores continued to decrease in this group during the 3 months of treatment (*P* < .01). After 12 treatments with acupuncture plus fire needle therapy, there was a significant improvement in the MGS of the affected hand (*P* < .01) and in both sections of the PRFEQ, particularly for pain (*P* < .01) and function (*P* < .01). We also observed a significant improvement in the components of the SF-36, particularly in the PCS (*P* < .01) and MCS (*P* < .05) at the end of treatment; this improvement continued in the acupuncture plus fire needle group during the 3 months of treatment (*P* < .01; Table 2).

**Table 2:**
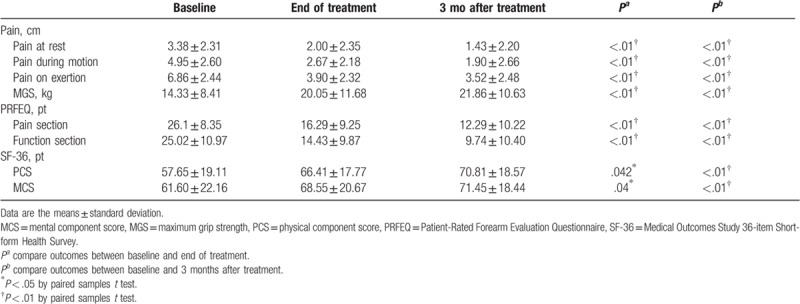
Outcomes at the end of treatment and 3 months after the completion of treatment with acupuncture plus fire needle group.

Within-group analyses revealed no significant differences in any of the outcome scores at the end of treatment in the acupuncture-only group (all *P* > .05), except in the MGS score for the affected hand (*P* < .01). Three months after completion of acupuncture, there were significant improvements in VAS pain scores at rest (*P* < .05) and during motion (*P* < .01) and exertion (*P* < .01). After completion of 3 months of acupuncture, there was still a significant improvement in the MGS of the affected hand (*P* < .01) and in both sections of the PRFEQ (*P* < .01). There was a significant change in the MCS (*P* < .05) and PCS (*P* < .01) sections of the SF-36 after 3 months of treatment in this group (Table 3).

**Table 3:**
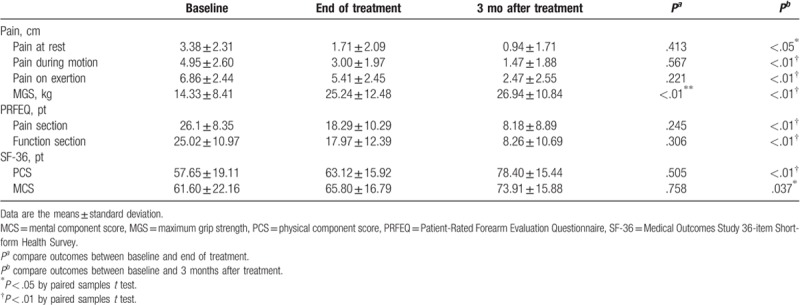
Outcomes at the end of treatment and 3 months after the completion of acupuncture group treatment.

There was no significant between-group difference in the mean VAS or MGS score for the affected hand or in the PRFEQ and SF-36 scores at baseline, at the end of treatment, or at follow-up 3 months later (*P* > .05). However, there was a tendency for better improvement in the short term in the acupuncture plus fire needle group than in the acupuncture group (Table 4).

**Table 4:**
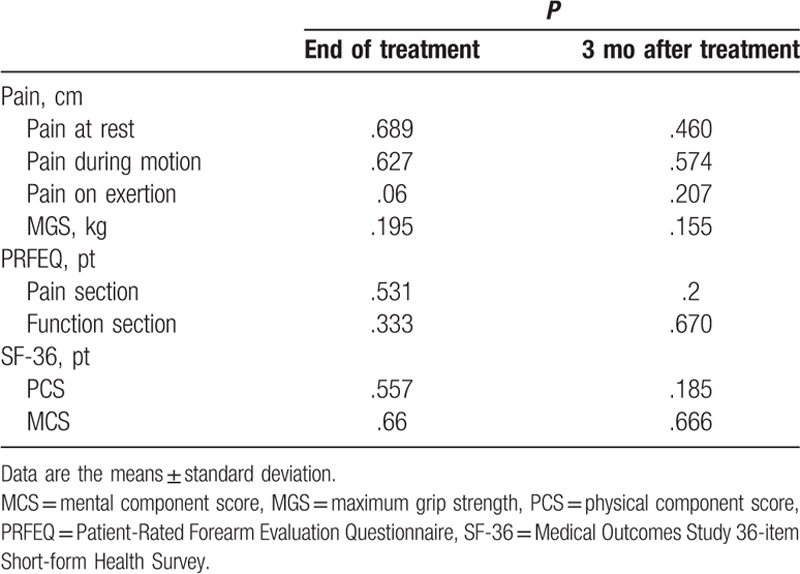
Comparisons of the outcomes at the end of treatment and 3 months after treatment, acupuncture plus fire needle group versus acupuncture group.

## 4 Discussion

We have compared the short-term and intermediate-term effects of acupuncture plus fire needle therapy on pain, grip strength, and quality of life with those of acupuncture alone in patients with LE.

Our acupuncture plus fire needle group showed significant improvements in VAS scores for elbow pain and in PRFEQ, MGS, and SF-36 scores at the end of treatment and 3 months after completion of treatment. These findings indicate that acupuncture plus fire needle therapy significantly improves pain and functional impairment in patients with LE in both the short term and intermediate term. Unlike acupuncture plus fire needling, acupuncture did not obviously reduce pain or functional impairment initially. Nevertheless, there was a significant increase in MGS scores in the acupuncture-only group after completion of treatment. Furthermore, symptoms abated and function improved more dramatically in the acupuncture group in the intermediate term. There was a trend of greater improvement in the VAS, PRFEQ, and SF-36 scores in the acupuncture group in the short term. Overall, there were clear decreases in the pain and functional impairment caused by LE in both study groups in the intermediate term.

Although some trials reported in the Chinese journals have demonstrated the efficacy of fire needle treatment for LE, to the best of our knowledge, there have been no relevant randomized controlled trials of this intervention reported in the English language. Several studies have reported an association between decreased grip strength and LE.^[24,25]^ Therefore, improvement in grip strength could indicate a positive treatment outcome.^[26]^ Acupuncture seemed to be effective in the short term in our study. We attribute the lack of significant objective improvement to the fact that the short-term effects of treatment were evaluated immediately after completion of 12 acupuncture treatments. Some patients may not have felt any subjective improvement at this time because acupuncture itself can induce mild local pain at the needling area. Although acupuncture only improved the MGS score, its effects on pain and functional outcomes after 3 months of treatment were better than those immediately following treatment. A systematic review reported that acupuncture not only has a good immediate analgesic effect but also that its effect is unremarkable in the long term.^[16]^ Ural et al^[27]^ recently performed a randomized controlled trial in which a group that received 4 weeks of acupuncture demonstrated significantly fewer symptoms and less common extensor tendon thickness on ultrasonography, and these findings were closely related to the severity of LE. In our study, we demonstrated that acupuncture was an effective treatment in the intermediate term for patients with LE. This finding is consistent with that of Grua et al,^[28]^ who investigated acupuncture and ultrasonography in a randomized trial of 40 patients with LE and found that improvements in pain and functional recovery were significantly different after treatment and at the 6-month follow-up in the treatment group. A study by Fink et al^[29]^ showed that the severity of symptoms was diminished after 10 treatments in their acupuncture (treatment) group and sham acupuncture (control) group; however, significantly more improvement was noted in the treatment group than in the control group at the 2-week follow-up visit. The difference was still significant at 2 months, at which time function was more improved in the treatment group.

Fire needle therapy was first described in the Yellow Emperor's Canon of Internal Medicine (Huángdì nèijīng, 475–221 BC) over 2000 years ago. A burning needle was the key modality in this treatment. The needle must be heated until it glows a red color; subsequently, the glowing needle is inserted. The heat of a fire needle dissipates rapidly, so the needle is not irritating, even though the body of needle remains in the skin and focuses the heat directly on a specific area.^[30]^ Hence, fire needle therapy stimulates acupoints and has a moxibustion effect simultaneously, with the effect of promoting the circulation of blood and eliminating blood stasis.^[31]^ In traditional Chinese medicine, fire needle therapy has the effect of warming and freeing the yang qi of the body and therefore repels wind and cold dampness in the meridian.^[32]^ The effect is correlated with the most common traditional Chinese medicine patterns of LE, that is, a wind-cold dampness pattern as well as qi stagnation and blood stasis pattern.^[33]^ Therefore, according to traditional Chinese medicine theory, fire needle therapy seems to be an appropriate treatment for LE.

In our study, the pain and functional impairment caused by LE was significantly decreased in the acupuncture plus fire needle group in the short term and the effect lasted well into the intermediate term. The pain was relieved more rapidly, albeit not to a statistically significantly extent, in the fire needle group than in the acupuncture group. These results are in agreement with the findings of Ding^[34]^ and Zhu and Zhu,^[35]^ who showed that the signs and symptoms of LE in patients who received acupuncture plus fire needle therapy were reduced in comparison with those in patients who received conventional acupuncture treatment.

This study had several limitations that need to be acknowledged. First, our study participants had had symptoms of tennis elbow for up to 2 months, so our findings cannot be generalized to patients with symptoms of longer duration. However, the impact of this limitation was difficult to assess because it was not possible to include a second control group that received no treatment for 3 months. Second, the study participants were recruited solely from outpatient clinics, which might have introduced a degree of selection bias. However, had we not used this recruitment strategy, the sample size would have been too small and the study groups would not have been of equal size. The short follow-up period and use of single blinding rather than double blinding were additional limitations.

We found preliminary evidence to support the use of acupuncture and acupuncture plus fire needle therapy in the treatment of LE over a 3-month period. After 3 months of treatment, the severity of pain and functional impairment were reduced in both study groups. Moreover, the decrease in pain and functional impairment in the acupuncture plus fire needle group was greater than that in the acupuncture group in the short term. However, there were no significant between-group differences in the intermediate-term outcomes, meaning that both treatment strategies could achieve significant outcomes in the intermediate term, and only the acupuncture plus fire needle therapy had a significant short-term effect.

## 5 Conclusion

The findings of this preliminary study indicate that acupuncture plus fire needle therapy may be effective in patients seeking improvement of LE in the short term and that 12 acupuncture sessions with or without fire needle therapy were effective for relieving pain and improving disability in the intermediate term in these patients. These preliminary findings confirm the feasibility of conducting a larger-scale randomized controlled trial that includes a placebo (or sham) group and a longer follow-up duration to evaluate the effects of this combination treatment for LE.

## Acknowledgments

The authors would like to extend their appreciation to all the patients who participated in this survey. We appreciate the assistance with the statistical analysis provided by the Biostatistics Center, Kaohsiung Chang Gung Memorial Hospital.

## Author contributions

KWB and CNL conceived and designed the study. CNL, JMS, and KWB recruited the study participants. KWB and CNL performed the intervention. TPH and KWB collected the patient data. SYW and CCC interpreted and analyzed the data. SYW and CJC drafted the manuscript. CEK and CNL critically reviewed and revised the manuscript. All authors read and approved the final manuscript.

**Conceptualization:** Cheng-Nan Lu, Kuo-Wei Bi.

**Data curation:** Cheng-Nan Lu, Jer-Ming Sheen, Tun-Pin Hsueh, Kuo-Wei Bi.

**Formal analysis:** Szu-Ying Wu, Ching-Chih Chen.

**Methodology:** Szu-Ying Wu, Cheng-Nan Lu, Kuo-Wei Bi.

**Project administration:** Cheng-Nan Lu, Kuo-Wei Bi.

**Supervision:** Cheng-Nan Lu, Kuo-Wei Bi.

**Writing – original draft:** Szu-Ying Wu, Chia-Jung Chung.

**Writing – review & editing:** Cheng-Nan Lu, Chun-En Kuo.
